# Invasive *Mycobacterium chimaera* Infections and Heater-Cooler Devices in Cardiac Surgery

**DOI:** 10.3201/eid2603.191818

**Published:** 2020-03

**Authors:** Rami Sommerstein, Barbara Hasse, Andreas F. Widmer

**Affiliations:** Bern University Hospital, Bern, Switzerland (R. Sommerstein); University Hospital Zurich, Zurich, Switzerland (B. Hasse);; Basel University Hospital, Basel, Switzerland (A.F. Widmer)

**Keywords:** *Mycobacterium chimaera*, surgical site infection, cardiac surgery, healthcare-associated infection, tuberculosis and other mycobacteria, equipment contamination, disease outbreaks, cardiac surgical procedures, risk assessment

**In Response:** We would like to thank Lamagni et al. (*1*) for including additional cases from the period of 2008–2014 and for recalculating the UK cumulative incidence risk by matching our assessment. We agree that the observed 3 times higher risk in Switzerland of *Mycobacterium chimaera* infection for a patient undergoing heart valve surgery cannot be explained unambiguously. The most likely explanation is indeed increased awareness of the risk in Switzerland compared with other countries, potentially improving likelihood of investigation for mycobacterial infection. We think that the early formation of a national and interdisciplinary working group (*M. chimaera* Task Force), which included representatives of the involved (para-)medical disciplines, authorities, and professional associations, has contributed greatly to raising this awareness.

We want to emphasize that approximately half of the patients in Switzerland were treated for suspected sarcoidosis or other rheumatic diseases. It was via the active case-finding mechanisms that these cases were identified. Furthermore, we would like to point out that, since the publication of our report (*2*), only 1 case known to the task force has been identified in Switzerland (in a patient whose surgery was performed in 2014). This finding could indicate that the formation of the national task force not only increased awareness but also ensured that the remaining infectious risk was reduced to a minimum within a very short time.
